# Chronic Orchitis, and Extirpation of Testicle

**Published:** 1862-07

**Authors:** L. D. Robinson

**Affiliations:** Groveland, Ind.


ARTICLE XXI.

### CHRONIC ORCHITIS, AND EXTIRPATION OF TESTICLE.

By L. D. ROBINSON, M.D., Groveland, Ind.

In the month of Dec. 1861, I was called upon to treat the case of Mr. C. aged about 30 years, of nervo-lymphatic temperament; short in stature and stoutly built, possessing naturally great power of constitution.

*History of Case:*—In the month of September, of same year, he received an injury, consisting in a severe bruise of the left testicle, while attempting to mount a vicious mule. The morning following the receipt of the injury, he arose from bed with testicle much swollen, hot, and very painful—creating considerable symptomatic fever. He applied to a Dr. for medical advice. The Dr. pronounced his case an *hydrocele*, and treated it as such: thrusting his trochar to the centre of the gland.

The result was, that the Doctor's treatment augmented the trouble, increased the inflammation and suffering. The patient then changed physicians, and the new Dr. treated the case by warm emollient poultices, locally, and anti-phlogistic treatment constitutionally.

By this treatment suppuration was prevented at the time, and the gland passed into the chronic form of orchitis, with much enlargement, induration, and discoloration. The patient's general health began to give way, at this time, and he gradually sank into a cachexy of constitution.

He left Illinois in the month of Nov. following, and came into the neighborhood where I was then practicing.

*Condition at the time of my first Examination:*—Testicle enlarged to about four times the natural size. Scrotum covering the gland of very dark purple color, and much thickened. On examining the gland by palpation it was found to be quite hard and resisting. Sensation in the organ very much diminished—indeed, hard pressure upon the gland produced very little suffering. Patient's general health very much depraved—extreme debility, accompanied by functional derangements of almost all the organs of the body. And here I will digress enough to relate some circumstances connected with the case that were quite significant:—About the time of my first visit to the case, the patient received a slight injury upon the back of the left hand, which merely removed the cuticle off a small surface, which first assumed the form of an indolent ulcer, but subsequently formed the nidus of a semi-malignant fungus growth, which attained to the size of a hen's egg; meanwhile resisting all and every form of local treatment employed in the case. I used nit. silver, nit. acid, nit. cupri, etc., etc., all without any good impression. About the same time, the great toe of the left foot became inflamed with an asthenic form of inflammation, passed on to suppuration and ulceration, and then a similar growth to that on the hand supervened. The toe, as the patient informed me, upon inquiry, had been severely affected by chilblain, a few years ago, which rendered it a weak point. The scrotum also had upon its surface a small abraded spot, which assumed the same form as the hand and toe.

My first impression was that the gland would have to be extirpated; but the patient prefering medical treatment for his relief, I adopted the following constitutional treatment, knowing that it would put him in a much better condition for an operation, than he was at the time of prescribing it, any how:

℞. Syrup sarsaparilla comp. ............℥iv.

Iodide potassa, ........................ʒiij.

Mix. Dose:—a teaspoonful once in four hours. Alternating with the above sulphs. quinia and morphia.

Locally, I blistered the scrotum, and applied unguentum mercuriale.

Upon this management, the patient's general health improved rapidly, for about six weeks, and the gland decreased to almost half its former size, and in two weeks more, to almost its normal size.

But about this time, the patient took a long and fatiguing walk, which was speedily followed by a return of the local difficulty, and that in turn seemed to reproduce the same form of constitutional trouble, but in a milder degree. The same form of treatment, with the alteration of substituting strychnia for the quinia, after being employed a sufficient length of time, and seeming to do but little good, caused me to think that the difficulty was assuming a malignant form, and that the gland would probably require extirpation. But here two questions came up, viz.: Had the trouble not already assumed a malignant form, and if so, would an operation afford permanent relief? Or would not the wound, that would be necessarily inflicted in the operation, assume the same form of the wound on the hand and the toe?

While indulging my thoughts in this quandary, I wrote Prof. N. S. Davis, detailing to him the case, and asking his advice. His advice was to extirpate "at once;" and then give the patient the tinct. cinchonæ, and bi-chloride hydrargyri, in proper proportions and at proper intervals.

I followed the Prof.'s advice, and proceeded to extirpate the gland in the following manner:—

The patient having been placed in the proper position, and brought fully under the influence of sulph. ether and chloroform, aa., by my brother, Dr. J. H. ROBINSON, I proceeded to remove the hair from about the groin and scrotum, and then, with bistoury in hand, I made an incision, beginning just below the external abdominal ring, and terminating at the lower extremity of the affected gland, running parallel with and close to the raphe or mesian line of the scrotum. I then made a second incision, beginning at the upper portion of the testicle, and running parallel with the first incision, including all that portion of the scrotum that was sufficiently diseased to demand removal. Found the scrotum and testicle morbidly adherent, and the tunics almost completely obliterated, so much so, that surgical anatomy afforded me no guide in the operation. I then proceeded to carefully dissect the gland loose from its attachments, which I found to be somewhat tedious and difficult, owing to the change that disease had wrought in the structures, and the hemorrhage that was occasioned by the wounding of the distal extremities of the spermatic arteries.

But after getting the gland ready to sever the cord, and remove the testicle, I adopted the novel plan of ligating the cord, with the accompanying arteries and nerves, all together, and severing them upwards. I then proceeded to dress the wound in the usual way.

Reaction came up finely, with no fever or secondary hemorrhage, and with but little pain. Gave patient $\frac{1}{2}$ gr. sulph. morphia, and in two hours repeated the dose, which was the only anodynes I found it necessary to employ in the case. The patient did well, and the wound healed rapidly and kindly, with but little inflammation or suppuration.

The patient was able to be on foot within a month's time, from the date of the operation. The general health improved finely, under the influence of Dr. DAVIS'S prescription, and the hand and toe became almost well, apparently.

This method of ligating I claim to be much better, safer, and quicker than the method required by our standard surgical authors. They instruct you to first incise the cord and blood-vessels, and ligate afterwards, meantime trusting the cord to the hands of an assistant, while you separate the arteries from the cord and nerves.

1st. I regard it very unsafe to trust the arteries to the grasp of an assistant, before you tie them, lest they slip away and are drawn through the ring into the abdomen, and there produce a fatal hemorrhage.

2d. I regard it as wholly unnecessary to subject the surgeon to the tedious and troublesome task of carefully separating the arteries from the cord, nerves, and muscular structure, and tieing them (the arteries) singly.

3d. I regard it just as safe, in every particular, and just as free from after suffering—much simpler, and less difficult to ligate arteries, cord, nerves, etc., all together, as it is to ligate the bloodvessels without including the spermatic nerves in the ligature. No more pain was inflicted in this case than there is in the usual operation of incising a nerve trunk, and the method of ligating, I am sure, is much easier than any other.

In conclusion, I would suggest that it is *possible* that this patient was laboring under a syphalitic taint of constitution; yet I have detailed the case as *it was*, and think it *very improbable*, as the character and veracity of the gentleman, together with his general appearance, rather discourages this conclusion.

